# Analysis of Mpox by Occupation and Industry in Seven U.S. Jurisdictions, May 2022–March 2023

**DOI:** 10.3390/ijerph21101317

**Published:** 2024-10-03

**Authors:** Matthew R. Groenewold, Marie A. de Perio, Kyle M. Moller, David Bui, Kayla Saadeh, William Still, Ie Meh, Antionette Lavender, Susan Soliva, Caitlin Fields, Brandi Hopkins, Angela K. Laramie, Pauline Harrington, Allen Stout, Cheri Levenson, Collin R. Morris, Paul D. Creswell, Hannah E. Segaloff, Nicholas J. Somerville, Chad H. Dowell, Lisa J. Delaney

**Affiliations:** 1 National Institute for Occupational Safety and Health, Centers for Disease Control and Prevention, Cincinnati, OH 45226, USA; matthew.groenewold@bsu.edu (M.R.G.); yxp3@cdc.gov (N.J.S.); 2 National Institute for Occupational Safety and Health, Centers for Disease Control and Prevention, Atlanta, GA 30345, USA; kyle.m.moller2.mil@health.mil (K.M.M.); crd7@cdc.gov (C.H.D.); lkd2@cdc.gov (L.J.D.); 3 California Department for Public Health, Richmond, CA 94804, USA; davidbui7@gmail.com (D.B.); kayla.saadeh@cdph.ca.gov (K.S.); 4 District of Columbia Department of Health, Washington, DC 20002, USA; will.still@dc.gov (W.S.); ie.meh@dc.gov (I.M.); 5 Georgia Department of Public Health, Atlanta, GA 30334, USA; antionette.lavender@dph.ga.gov; 6 Massachusetts Department of Public Health, Boston, MA 02130, USAcaitlin.fields@gmail.com (C.F.); brandi.hopkins@mass.gov (B.H.); angela.laramie@mass.gov (A.K.L.); 7 Michigan Department of Health and Human Services, Lansing, MI 48909, USA; harringtonp4@michigan.gov (P.H.); stouta4@michigan.gov (A.S.); 8 Washington State Department of Health, Tumwater, WA 98501, USA; cheri.levenson@doh.wa.gov; 9 Wisconsin Department of Health Services, Madison, WI 53703, USA; collin.morris@dhs.wisconsin.gov (C.R.M.); paul.creswell@dhs.wisconsin.gov (P.D.C.); qdz0@cdc.gov (H.E.S.); 10 Centers for Disease Control and Prevention, Atlanta, GA 30329, USA

**Keywords:** mpox, monkeypox, occupation, industry, epidemic, surveillance

## Abstract

During responses to outbreaks, the collection and analysis of data on employed case patients’ industry and occupation are necessary to better understand the relationship between work and health outcomes. The occurrence of mpox by occupation and industry has not previously been assessed in the context of the 2022 outbreak. We analyzed employment data from 2548 mpox cases reported to the U.S. Centers for Disease Control and Prevention from surveillance systems in seven U.S. jurisdictions and population-based reference data on employment patterns from the U.S. Bureau of Labor Statistics to describe the differential proportionate distribution of cases across occupation and industry groups using the proportionate morbidity ratio. In gender-specific analyses, we found that men employed in certain occupations and industries had a higher relative risk of mpox than others. While occupational transmission cannot be ruled out, it is more likely that individuals with personal and behavioral risk factors for mpox were more likely to work in these occupations and industries. This analysis provides an example of collecting and analyzing occupation and industry data in case reports to understand possible differences in risk by occupation and industry in infectious disease outbreak investigation and help inform resource allocation, messaging, and response.

## 1. Introduction

The global mpox outbreak began in May 2022 and had affected more than 93,000 patients worldwide by December 2023 [1]. Monkeypox virus, the causative agent of mpox, typically spreads through close physical contact with the lesions, scabs, body fluids, or respiratory secretions of a person with an active Monkeypox virus infection [2]. The risk of occupational mpox transmission in the U.S. workforce overall is likely low. However, the occurrence of mpox by occupation and industry has not been assessed in the context of the 2022 outbreak. While the risk appears to be low [1], occupationally acquired mpox has been reported in U.S. healthcare personnel, mostly associated with needlestick injuries [3,4]. Occupational transmission in other work settings has not been documented in the United States during the current outbreak. However, because occupational data are not captured in national mpox surveillance [5], there is no mechanism for identifying excess risk in occupation or industry groups at the national level.

Even if the absolute risk is low, the relative risk of occupational mpox transmission may differ by occupation or industry. Workers whose jobs involve physical contact with other people, such as healthcare personnel, massage therapists, barbers, beauticians, tattoo artists, and sex workers, may be at increased risk of acquiring or transmitting mpox.

Differences in the occupation- or industry-specific occurrence of mpox may be due to (1) differences in the risk of occupational acquisition (i.e., infection while working resulting from occupational exposure) or (2) the risk profiles of these worker subpopulations resulting from their demographic composition. Social or economic selection mechanisms may result in the clustering of workers with preexisting demographic or behavioral risk factors for infection such as men who have sexual contact with men, independent of any occupational exposures, within certain occupations or industries. Thus, the identification of higher-risk occupations or industries is important for the mitigation of both the risk of workers being infected on the job and of workers transmitting the virus to clients, customers, or the public if they work while infected. The identification of higher risk occupation and industry groups may also be useful for the targeting of public health prevention messaging and interventions.

We described the distribution of mpox cases and estimated the differential risk of mpox by occupation and industry among workers in seven U.S. jurisdictions that collected occupational data in their mpox surveillance systems during the 2022–2023 outbreak.

## 2. Materials and Methods

We analyzed case data reported to CDC from surveillance systems in seven U.S. jurisdictions and population-based reference data on employment patterns from the Bureau of Labor Statistics (BLS). We described the differential proportionate distribution of mpox cases across occupation and industry groups, relative to the expected distribution in the source population, using the proportionate morbidity ratio (PMR).

### 2.1. Case Data

As of 23 June 2022, when mpox was designated by the Council of State and Territorial Epidemiologists (CSTE) as a nationally notifiable disease, data on the clinical and demographic characteristics of probable or confirmed mpox cases were reported by all U.S. jurisdictions to the CDC using data elements included on CDC’s mpox short case report form (sCRF) [5]. This included retrospective reporting of outbreak-associated cases that occurred before 23 June 2022. Because occupational variables were not included on the sCRF, CDC’s National Institute for Occupational Safety and Health (NIOSH) partnered with six states—California, Georgia, Massachusetts, Michigan, Washington, and Wisconsin—and the District of Columbia, all of which collected occupational variables from some or all patients with confirmed or probable mpox identified in their jurisdictions, to generate a sample of mpox cases that could be used for occupational analyses. All available occupational data for cases in these seven jurisdictions occurring through March 23, 2023, along with the associated case identification numbers, were transmitted directly to NIOSH and were then linked to the cases’ full surveillance records contained in the CDC surveillance database for analysis. The period and scope of occupational data collection varied by jurisdiction. Details on the timing and sampling methodology for each jurisdiction’s collection of occupational data are presented in Supplementary Table S1.

Information on case patients’ current occupation and industry for their primary job—the job at which they worked the most hours—was solicited from all or a subset of currently employed cases, depending on state or local procedures for case investigation. Occupation is a person’s job or job title, while industry is the type of business in which a person works. In six of the seven jurisdictions, occupation and industry data were collected in the form of narrative descriptions captured in free-text fields and subsequently coded to 2010 Census occupation and 2012 Census industry codes using the NIOSH Industry and Occupation Computerized Coding System (NIOCCS) [6]. Five jurisdictions performed the coding themselves using the NIOCCS web application and transmitted both free-text descriptions and corresponding Census codes to NIOSH, while two jurisdictions provided text descriptions only, and NIOSH performed the coding.

All original occupation codes were crosswalked to 2018 Census occupation codes and then grouped into 23 occupation groups corresponding to the BLS Current Population Survey (CPS). All original industry codes were crosswalked to 2017 Census industry codes and then grouped into 22 industry groups corresponding to the BLS CPS [7].

Records for case patients, who were not in the labor force or unemployed at illness onset, who did not provide occupational data, or for whom a valid Census occupation or industry code could not be assigned, were excluded from the analyses.

### 2.2. Statistical Analyses

For each industry and occupation group, we described the proportional distribution of cases across categories of four demographic variables: age group, gender, race, and ethnicity. Because detailed information on gender identity (“cisgender man”, “transgender man”, “cisgender woman”, “transgender woman”, and “another gender identity”) was available for most case patients, we analyzed gender rather than sex. Case patients whose gender identities were unknown or missing were analyzed based on sex assigned at birth when available.

PMRs were calculated as the ratio of the total observed number of cases in our study sample for each occupation and industry group to an expected number. The expected number for each group was calculated by applying jurisdiction-specific occupation and industry employment proportions to the total number of cases in each respective jurisdiction’s study sample and summing those numbers across the seven jurisdictions, accounting for the differing contribution of cases from each. Jurisdiction-specific occupation and industry employment proportions were calculated by averaging monthly employment estimates from the CPS, without seasonal adjustment, from the first through to the last months in which mpox cases that the jurisdiction contributed to the analytic sample had their onset. Because the CPS does not include military personnel, we used data on the number of active-duty military personnel residing in each jurisdiction from the DoD Defense Manpower Data Center [8].

We also calculated gender-specific PMRs by occupation and industry. Because reference data on employment distributions by occupation and industry in the source population were only available by sex, we dichotomized the gender variable, including cis- and transgender people in the larger gender category (man or woman) with which they identified. Cases for which gender data were unknown or missing were analyzed based on sex assigned at birth when available. Cases for which both gender and sex assigned at birth were unknown and case patients who identified with another gender identity (i.e., other than man or woman) were excluded from the gender-stratified PMR analyses. Because sex-stratified data on military population sizes were not available by jurisdiction, we applied the known overall sex distribution of military servicemembers (17.5% female for all DoD service branches and 15% for the U.S. Coast Guard) to each jurisdiction’s military population to produce sex-specific estimates [8].

PMRs greater than or less than 1 indicate higher or lower than expected proportions of events within that occupation or industry group, respectively. We calculated 99% confidence intervals for all PMRs using the Vandenbroucke method [9]. PMRs were considered statistically significant if the null value, 1, was excluded from the 99% confidence interval. We used 99% rather than 95% confidence intervals to partially compensate for the increased type I error risk associated with multiple testing. Analyses were conducted using Microsoft Excel for Microsoft Office 365 and Epi Info 7.

### 2.3. Ethics Approval

This activity was reviewed by CDC, deemed not research, and was conducted consistent with applicable federal law and CDC policy (45 C.F.R. part 46, 21 C.F.R. part 56; 42 U.S.C. §241(d); 5 U.S.C. §552a; 44 U.S.C. §3501 et seq).

## 3. Results

Of the 9900 total mpox cases reported to the CDC from our seven participating jurisdictions as of 23 March 2023, corresponding records representing the attempted collection of occupational data were transmitted to NIOSH for 6854 (69.2%), which included those who reported not being in the labor force, being unemployed, or who declined to provide occupational data during case investigation. The jurisdiction-specific proportions of the total number of mpox cases from which occupational information was solicited ranged from 1.9% to 99.4%. Of these 6,854 cases, 2548 (37.2%) had either a valid occupation or industry code assigned. Of those, 2127 (83.5%) and 2269 (89.1%) had valid occupation and industry codes assigned, respectively. Details on the jurisdiction-specific numbers and proportions of all mpox cases from which occupational data were solicited and for which valid occupation or industry codes were assigned are presented in Supplementary Table S2.

The distributions of mpox cases in our sample across age group by occupation group and industry sector are shown in Table 1 and Table 2.

Consistent with national case demographics, nearly all the cases—2463 (96.7%)—in our overall sample were among cisgender men. Cisgender women accounted for 2.1% of cases, while transgender men, transgender women, and people with another gender identity each accounted for less than 1% of cases. Also consistent with national case demographics, over two-thirds (71.5%) of the cases in our overall sample were among people aged between 25 and 44 years. Slightly over half (51.9%) of the cases in our sample were among people who identified as White. People who identified as Black or African American accounted for 18.4% of the sample, with the remaining 29.7% of cases occurring among people of another race or whose race was not known. People who identified as being of Hispanic or Latinx ethnicity accounted for 37.4% of the cases in our overall sample.

Among the 2,127 cases with valid occupation codes, the most frequently reported occupation group was transportation and material moving occupations (n = 214, 10.1%), followed by sales and related occupations (n = 212, 10.0%), office and administrative support (n = 206, 9.7%), management (n = 197, 9.3%), and food preparation and related (n = 194, 9.1%) occupations. Among the 2,269 cases with valid industry codes, the most frequently reported industry group was the healthcare and social assistance industry (n = 407, 17.9%), followed by the accommodation and food services (n = 278, 12.3%); professional, scientific, and technical services (n = 230, 10.1%); retail trade (n = 228, 10.0%); and other services (except private households) (n = 165, 7.3%).

**Figure 1:**
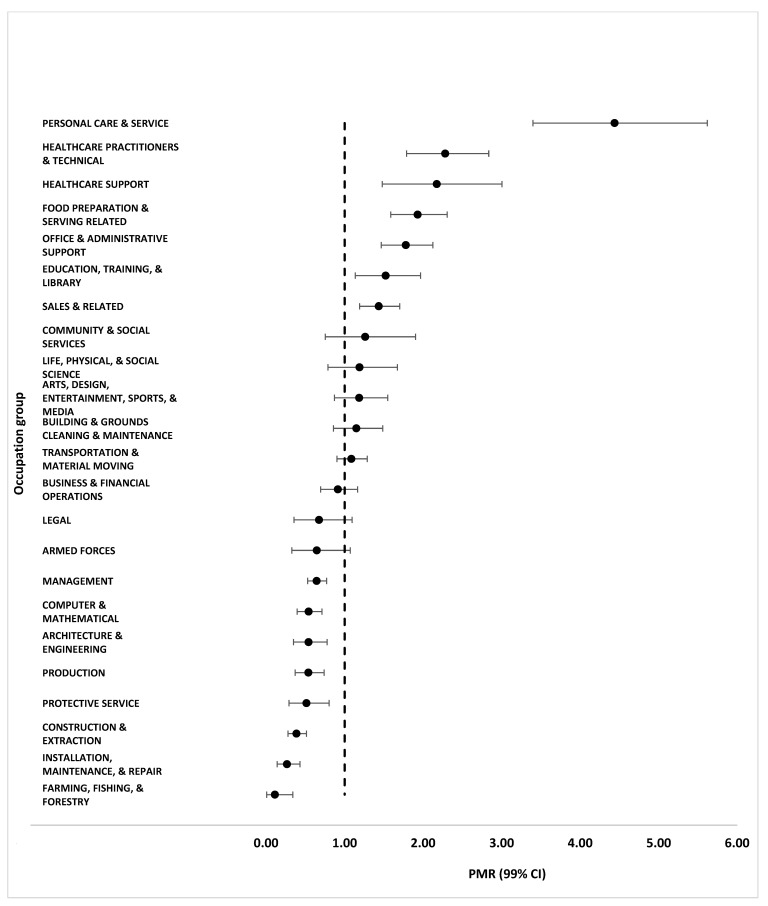
Proportionate morbidity ratios (PMRs) and 99% confidence intervals (CIs) by occupation group in a sample of employed mpox cases among men from seven U.S. jurisdictions, May 2022–March 2023.

**Figure 2:**
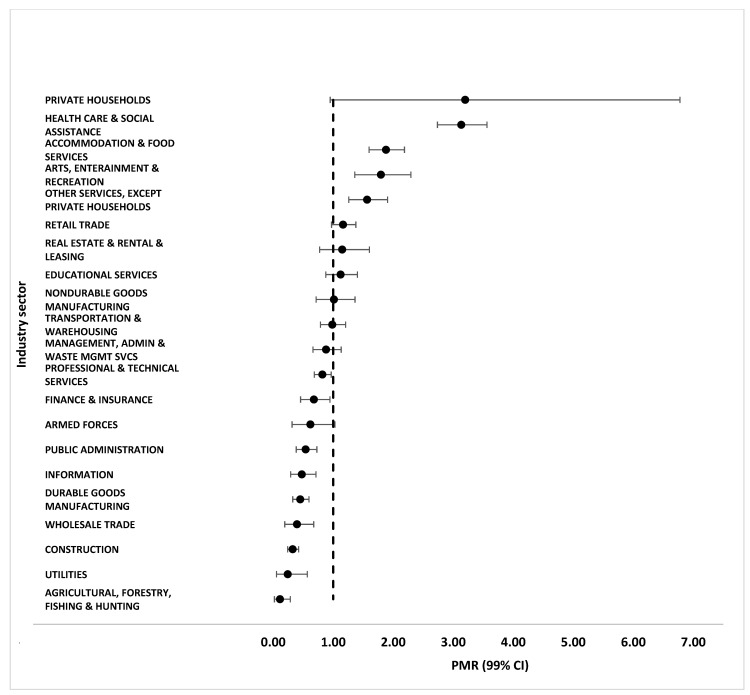
Proportionate morbidity ratios (PMRs) and 99% confidence intervals (CIs) by industry sector in a sample of employed mpox cases among men from seven U.S. jurisdictions, May 2022–March 2023.

PMRs by occupation group and their associated 99% confidence intervals, for men only, are shown in Figure 1. PMRs by industry group for men only are displayed in Figure 2. In the overall sample, significantly higher than expected proportions of cases occurred among people employed in four occupation and five industry groups, and significantly lower than expected proportions occurred in seven occupation and ten industry groups (Supplementary Tables S3 and S4). However, because our sample of cases overwhelmingly comprised men, we considered the gender-specific PMRs to be the more reliable. We emphasized those results, with special attention to the significantly elevated PMRs as indicators of potentially higher-risk occupation and industry groups. Tables of PMRs by all genders are included in the Supplementary Tables S3 and S4.

In gender-specific analyses by occupation, significantly higher than expected proportions of cases occurred among men employed in personal care and service (PMR 4.44, 99% CI 3.40–5.62); healthcare practitioners and technicians (PMR 2.28, 99% CI 1.79–2.83); healthcare support (PMR 2.17, 99% CI 1.48–3.00); food preparation and serving-related (PMR 1.93, 99% CI 1.59–2.31); office and administrative support (PMR 1.78, 99% CI 1.47–2.12); education, training, and library (PMR 1.52, 99% CI 1.14–1.97); and sales and related (PMR 1.44, 99% CI 1.19–1.70) occupations. Personal care service occupations include workers who attend to clients’ beauty, fitness, and other needs. Food preparation and serving-related occupations include workers who make and provide food and drink to customers. Sales and related occupations include workers who sell goods and services or connect buyers with sellers in a specific market, such as real estate or securities. Significantly lower than expected proportions of cases also occurred among men employed in eight occupation groups, as indicated in Figure 1.

In gender-specific analyses by industry, significantly higher than expected proportions of cases occurred among men employed in the healthcare and social assistance (PMR 3.14, 99% CI 2.74–3.56); accommodation and food services (PMR 1.88, 99% CI 1.60–2.19); arts, entertainment, and recreation (PMR 1.80, 99% CI 1.36–2.29); and other service (except private households) (PMR 1.57, 99% CI 1.26–1.91) industries. Significantly lower than expected proportions of cases also occurred among men employed in ten industry groups, as indicated in Figure 2.

Significant differences in the observed versus expected proportions of cases by occupation and industry were not identified among women due to the extremely small number of women in the sample.

## 4. Discussion

In the United States, 2022 outbreak data suggest that the community transmission of mpox has occurred mainly in the context of sexual or close intimate contact [10,11]. Our findings suggest that men employed in certain occupations and industries had a higher relative risk of mpox than others. However, since workplace transmission has not been identified as a significant mode of transmission in this outbreak, it is more likely that individuals with personal and behavioral risk factors for mpox were more likely to work in these occupations and industries (e.g., in the context of the current outbreak, men who have sexual contact with other men). To the extent that this hypothesis holds true, the effect of occupation and industry on the risk of mpox should be seen as compositional rather than contextual [12].

Occupations in the personal care and service category had the highest PMR. This is most likely a function of higher proportions of cisgender men with personal risk factors working in these occupations. However, it is possible there is an element of occupational risk due to the nature of the work, where skin-to-skin contact is necessary. Broad occupations that fall within this category included personal appearance workers and personal care aides. Similarly, the industry category of other services, except private households, also had a higher PMR. Tables showing the most frequently reported detailed occupations and industries for identified high-risk occupation and industry groups are included in Supplementary Tables S5–S7.

During the 2022 outbreak, there was interest in the unique occupational risks of mpox transmission and a focus of prevention measures on sex work as an industry [13]. In Germany and Italy, among the first reported cases of mpox in May 2022 were two men who were reported to be involved in sex work [14,15]. In our sample of 2127 workers with known occupations, only 12 case patients were reported to have occupations that would fit under the term “sex worker.” Occupations that would fit under the umbrella term “sex worker” could include occupations, such as prostitutes, escorts, strippers, adult entertainers, and adult film actors. Sex worker does not have a standard occupational code, and these occupations may fit under the categories of personal care and service occupations or under the arts, design, entertainment, sports, and media occupations. The difference in classification would depend mainly on whether the worker is paid to engage in sex or intimate physical contact with a customer or client directly versus being paid to engage in nudity, unpartnered sex, or sex with other sex workers—either as part of a live performance or to be captured in images, video, or film. All 12 of the identified sex workers in our sample were classified in the personal care and service category. Similarly, the sex work industry may encompass multiple categories, including arts, entertainment, and recreation; accommodation and food services; and other services. Our findings may be an underestimate of cases among these workers as our analysis included data on primary employment. We did not account for secondary or side employment, which may have caused us to miss cases in this occupational group. Further, some sex workers may have chosen not to disclose their occupations.

Interestingly, all the categories comprising healthcare personnel (HCP)—including practitioner, technical, and support occupations, as well as the healthcare and social assistance industry—had elevated PMRs. As of May 9, 2023, 1,235 cases had been reported among HCP worldwide; most were reported to be infected in the community and not at work [1]. Only a few case reports of occupational transmission of mpox from patients to HCP in healthcare settings worldwide in this outbreak have been reported [3,16,17,18]. These have largely been associated with needlestick injuries from unroofing or aspirating lesions, a practice which is not recommended, and some HCP received orthopox vaccination as part of post-exposure prophylaxis [2,17,19,20,21,22]. While occupational mpox transmission has been reported to occur in previous outbreaks, this has mainly occurred in countries in sub-Saharan Africa, where infection prevention and control practices are often inadequate [23]. Nevertheless, the risk of transmission from infected patients to HCP in the United States is very low [24,25]. While this analysis did find an increased PMR among certain occupational groups, whether or not occupational exposure was the cause of disease in these instances could not be assessed; based on other published data on the risk of occupationally acquired mpox, it is more likely that non-occupational exposures were the primary driver of the elevated PMR in these groups.

While occupational transmission has not been a prominent feature of the 2022 outbreak, it has been described in previous outbreaks and could be a feature in the future [26]. In addition to healthcare personnel, it has been long recognized that mpox is a zoonotic disease that confers occupational risks to those who work with certain exotic animal species. In the 2003 outbreak of prairie-dog-associated mpox, occupationally acquired infections occurred among veterinary staff, pet store employees, and animal distributors [27,28].

The findings in this report are subject to certain limitations. First, this report reflects a passively collected convenience sample of seven U.S. jurisdictions; this limits the generalizability of the distribution of cases by occupation and industry to all jurisdictions nationwide. Second, occupation and industry were not able to be ascertained for all currently employed cases in any of the participating jurisdictions, either because this information was only solicited from a subset of cases patients, because significant proportions of case patients did not provide occupational information, or both. Therefore, the true size of the source population for our analytic dataset was not known. There was also the possibility of differential bias in the reporting of occupations, given workers in some occupations are more likely to be forthcoming about their occupation than others. Additionally, because only probable or confirmed cases were interviewed and included in surveillance systems, a sample of non-cases was not available for comparison, requiring the approximation of occupation- and industry-specific relative risks using PMRs. Third, while we controlled for state and gender in our PMR calculations, we were not able to control for sexual orientation or behavioral or other individual characteristics associated with mpox risk in the current outbreak, such as men who have sexual contact with other men, as robust population prevalence estimates are not available. To the extent that such risk factors are also associated with occupation or industry, they could confound the relationship between these latter variables and mpox risk, as mentioned above. Finally, we did not determine if any of the case patients had occupationally acquired mpox, as this was outside the scope of our study. Thus, these findings should not be used to make any decisions about occupational groups and mpox vaccination prioritization.

During responses to outbreaks and pandemics, there is an ongoing need for the collection of industry and occupation data to better understand the relationship between work and health outcomes [29]. These data can be valuable for public health practitioners and industry and labor leaders to inform sector-specific policies, provide tailored recommendations on mitigation measures, develop effective vaccine strategies, communicate risks to workers, and reduce work-related health disparities. While workplace transmission is not a major driver of this 2022 global mpox outbreak, these data may also be useful in identifying future workplace-associated outbreaks. The collection of work information at the case level as part of public health surveillance is necessary to detect occupations and industries that may be disproportionately affected.

To improve data collection in surveillance systems, NIOSH recommends that occupational questions should be standardized, information on both industry and occupation should be collected, and data should be analyzed with standard coding schemes to monitor disease trends in specific industries or occupations and protect workers’ health [30]. Outbreak-specific variables for identifying putative high-risk occupations not well captured in standard coding schemes, such as sex workers, should also be included in case investigation forms.

## 5. Conclusions

While occupational transmission has not been a prominent feature of the 2022 outbreak, our analysis suggests that the distribution of mpox cases may be higher in men employed in certain occupations and industries. It is possible that individuals with personal and behavioral risk factors for mpox may be more likely to work in these occupations and industries. Nevertheless, this analysis provides an example of collecting occupation and industry data in case reports and analyzing that data in order to identify groups that are disproportionately affected in infectious disease outbreak investigation. Even if the elevated PMRs for certain industries and occupations in this outbreak are not due to occupational transmission, such collection and analyses may be useful in understanding occupational risk during future outbreak responses and can help inform resource allocation, messaging, and response.

## Figures and Tables

**Table 1:** Distribution of age group by occupation group in sample of employed mpox cases from seven U.S. jurisdictions, May 2022–March 2023 (n = 2548).

	Age Group						
	Total	<25 yrs	25–34 yrs	35–44 yrs	45–54 yrs	55–64 yrs	65+ yrs
	No. Cases	No. (%)	No. (%)	No. (%)	No. (%)	No. (%)	No. (%)
Management	197	6 (3.0)	71 (36.0)	73 (37.1)	30 (15.2)	16 (8.1)	1 (0.5)
Business and financial operations	102	4 (3.9)	42 (41.2)	33 (32.4)	12 (11.8)	9 (8.8)	2 (2.0)
Computer and mathematical	79	2 (2.5)	38 (48.1)	26 (32.9)	12 (15.2)	1 (1.3)	0 (0.0)
Architecture and engineering	42	4 (9.5)	20 (47.6)	9 (21.4)	5 (11.9)	3 (7.1)	1 (2.4)
Life, physical and social science	48	3 (6.3)	18 (37.5)	16 (33.3)	9 (18.8)	1 (2.1)	1 (2.1)
Community and social service	33	5 (15.2)	16 (48.5)	6 (18.2)	6 (18.2)	0 (0.0)	0 (0.0)
Legal	23	0 (0.0)	10 (43.5)	10 (43.5)	2 (8.7)	1 (4.3)	0 (0.0)
Education, training and library	94	4 (4.3)	45 (47.9)	23 (24.5)	14 (14.9)	6 (6.4)	2 (2.1)
Arts, design, entertainment, sports and media	81	2 (2.5)	35 (43.2)	29 (35.8)	10 (12.3)	4 (4.9)	1 (1.2)
Healthcare practitioners and technical	135	7 (5.2)	52 (38.5)	44 (32.6)	23 (17.0)	8 (5.9)	1 (0.7)
Healthcare support	58	7 (12.1)	28 (48.3)	11 (19.0)	10 (17.2)	2 (3.4)	0 (0.0)
Protective service	27	7 (25.9)	10 (37.0)	6 (22.2)	4 (14.8)	0 (0.0)	0 (0.0)
Food preparation and serving related	194	30 (15.5)	87 (44.8)	53 (27.3)	17 (8.8)	7 (3.6)	0 (0.0)
Building and grounds cleaning and maintenance	90	8 (8.9)	27 (30.0)	36 (40.0)	17 (18.9)	2 (2.2)	0 (0.0)
Personal care and service *	113	9 (8.0)	39 (34.5)	37 (32.7)	18 (15.9)	8 (7.1)	2 (1.8)
Sales and related	212	32 (15.1)	92 (43.4)	53 (25.0)	27 (12.7)	8 (3.8)	0 (0.0)
Office and administrative support	206	20 (9.7)	82 (39.8)	74 (35.9)	23 (11.2)	7 (3.4)	0 (0.0)
Farming, fishing and forestry	3	0 (0.0)	1 (33.3)	2 (66.7)	0 (0.0)	0 (0.0)	0 (0.0)
Construction and extraction	72	7 (9.7)	26 (36.1)	19 (26.4)	14 (19.4)	6 (8.3)	0 (0.0)
Installation, maintenance and repair	22	3 (13.6)	7 (31.8)	6 (27.3)	5 (22.7)	1 (4.5)	0 (0.0)
Production	61	7 (11.5)	21 (34.4)	15 (24.6)	14 (23.0)	3 (4.9)	1 (1.6)
Transportation and material moving	214	20 (9.3)	83 (38.8)	65 (30.4)	33 (15.4)	12 (5.6)	1 (0.5)
Armed Forces	21	2 (9.5)	15 (71.4)	4 (19.0)	0 (0.0)	0 (0.0)	0 (0.0)
Missing occupation	421	36 (8.6)	169 (40.1)	139 (33.0)	56 (13.3)	19 (4.5)	2 (0.5)
**Total**	2548	225 (8.8)	1034 (40.6)	789 (31.0)	361 (14.2)	124 (4.9)	15 (0.6)

* 12 workers in this category self-identified as sex workers.

**Table 2:** Distribution of age group by industry sector in sample of employed mpox cases from seven U.S. jurisdictions, May 2022–March 2023 (n = 2548).

Industry Sector	Age Group						
	Total	<25 yrs	25–34 yrs	35–44 yrs	45–54 yrs	55–64 yrs	65+ yrs
	No. Cases	No. (%)	No. (%)	No. (%)	No. (%)	No. (%)	No. (%)
Agriculture, forestry, logging, fishing and hunting	5	1 (20.0)	1 (20.0)	3 (60.0)	0 (0.0)	0 (0.0)	0 (0.0)
Mining	0	0 (0.0)	0 (0.0)	0 (0.0)	0 (0.0)	0 (0.0)	0 (0.0)
Construction	84	8 (9.5)	31 (36.9)	23 (27.4)	16 (19.0)	6 (7.1)	0 (0.0)
Durable goods manufacturing	76	10 (13.2)	29 (38.2)	17 (22.4)	17 (22.4)	2 (2.6)	1 (1.3)
Nondurable goods manufacturing	67	8 (11.9)	24 (35.8)	17 (25.4)	13 (19.4)	5 (7.5)	0 (0.0)
Wholesale trade	18	3 (16.7)	5 (27.8)	6 (33.3)	3 (16.7)	1 (5.6)	0 (0.0)
Retail trade	228	37 (16.2)	96 (42.1)	62 (27.2)	26 (11.4)	6 (2.6)	1 (0.4)
Transportation and warehousing	149	11 (7.4)	57 (38.3)	50 (33.6)	23 (15.4)	8 (5.4)	0 (0.0)
Utilities	6	0 (0.0)	1 (16.7)	3 (50.0)	2 (33.3)	0 (0.0)	0 (0.0)
Information	36	1 (2.8)	18 (50.0)	14 (38.9)	2 (5.6)	1 (2.8)	0 (0.0)
Finance and insurance	52	2 (3.8)	19 (36.5)	22 (42.3)	6 (11.5)	2 (3.8)	1 (1.9)
Real estate and rental and leasing	52	2 (3.8)	17 (32.7)	19 (36.5)	7 (13.5)	7 (13.5)	0 (0.0)
Professional, scientific and technical services	230	10 (4.3)	111 (48.3)	70 (30.4)	25 (10.9)	10 (4.3)	4 (1.7)
Management, admin support and waste mgmt svcs	93	12 (12.9)	32 (34.4)	31 (33.3)	12 (12.9)	5 (5.4)	1 (1.1)
Educational services	124	9 (7.3)	55 (44.4)	27 (21.8)	24 (19.4)	7 (5.6)	2 (1.6)
Health care and social assistance	407	28 (6.9)	163 (40.0)	132 (32.4)	65 (16.0)	17 (4.2)	2 (0.5)
Arts, entertainment and recreation	101	7 (6.9)	43 (42.6)	33 (32.7)	10 (9.9)	7 (6.9)	1 (1.0)
Accommodation and food services	278	42 (15.1)	118 (42.4)	83 (29.9)	25 (9.0)	10 (3.6)	0 (0.0)
Private households	9	1 (11.1)	1 (11.1)	2 (22.2)	3 (33.3)	1 (11.1)	1 (11.1)
Other services, except private households	165	12 (7.3)	58 (35.2)	55 (33.3)	31 (18.8)	8 (4.8)	1 (0.6)
Public administration	68	4 (5.9)	23 (33.8)	27 (39.7)	7 (10.3)	7 (10.3)	0 (0.0)
Armed forces	21	2 (9.5)	15 (71.4)	4 (19.0)	0 (0.0)	0 (0.0)	0 (0.0)
Missing industry	279	15 (5.4)	117 (41.9)	89 (31.9)	44 (15.8)	14 (5.0)	0 (0.0)
**Total**	2548	225 (8.8)	1034 (40.6)	789 (31.0)	361 (14.2)	124 (4.9)	15 (0.6)

## Data Availability

The public health surveillance data that support the findings of this study are available from the seven participating U.S. jurisdictions, but restrictions apply to the availability of these data, which were used by agreement with the jurisdictions for the current study and so are not publicly available. The data are, however, available from the authors upon reasonable request and with the permission of the relevant jurisdictions.

## Supplementary Materials

The following supporting information can be downloaded at: https://www.mdpi.com/article/10.3390/ijerph21101317/s1, Table S1: Occupational data collection details for participating states; Table S2: Breakdown of state-specific contributions of mpox cases to the analytic sample; Table S3: Proportionate morbidity ratios (PMRs) and 99% confidence intervals (CIs) by occupation group and gender in a sample of employed mpox cases from seven U.S. jurisdictions, May 2022–March 2023; Table S4: Proportionate morbidity ratios (PMRs) and 99% confidence intervals (CIs) for by industry group and gender in a sample of employed mpox cases from seven U.S. jurisdictions, May 2022–March 2023; Table S5: Most frequently reported detailed occupations within identified high-risk occupation groups in a sample of employed mpox cases among men from seven U.S. jurisdictions, May 2022–March 2023; Table S6: Most frequently reported detailed occupations within identified high-risk industry sectors in a sample of employed mpox cases among men from seven U.S. jurisdictions, May 2022–March 2023; Table S7: Most frequently reported detailed industries in identified high-risk industry sectors in a sample of employed mpox cases among men from seven U.S. jurisdictions, May 2022–March 2023.

## References

[1] World Health Organization 2022-23 Mpox (Monkeypox) Outbreak: Global Trends. 25 January 2024. https://worldhealthorg.shinyapps.io/mpx_global/.

[2] World Health Organization Multi-Country Outbreak of Mpox: External Situation Report 21. 27 April 2023. https://www.who.int/publications/m/item/multi-country-outbreak-of-mpox--external-situation-report-21---27-april-2023.

[3] Mendoza R., Petras J.K., Jenkins P., Gorensek M.J., Mableson S., Lee P.A., Carpenter A., Jones H., de Perio M.A., Chisty Z. (2022). Monkeypox Virus Infection Resulting from an Occupational Needlestick—Florida, 2022. MMWR Morb. Mortal. Wkly. Rep..

[4] Alarcón J., Kim M., Balanji N., Davis A., Mata F., Karan A., Finn L.E., Guerrero A., Walters M., Terashita D. (2023). Occupational Monkeypox Virus Transmission to Healthcare Worker, California, USA, 2022. Emerg. Infect. Dis..

[5] Centers for Disease Control and Prevention Mpox: Case Reporting Recommendations for Health Departments. 1 September 2023. https://www.cdc.gov/mpox/php/case-reporting/.

[6] Centers for Disease Control and Prevention Welcome to the NIOSH Industry and Occupation Computerized Coding System (NIOCCS). 13 December 2022. https://csams.cdc.gov/nioccs/.

[7] US Census Bureau Industry and Occupation Classification. 8 October 2021. https://www.census.gov/programs-surveys/cps/technical-documentation/methodology/industry-and-occupation-classification.html.

[8] Governing 2021 Military Active Duty Personnel, Civilians by State. 1 February 2022. https://www.governing.com/now/2021-military-active-duty-personnel-civilians-by-state.

[9] Vandenbroucke J.P. (1982). A shortcut method for calculating the 95 per cent confidence interval of the standardized mortality ratio. Am. J. Epidemiol..

[10] Philpott D., Hughes C.M., Alroy K.A., Kerins J.L., Pavlick J., Asbel L., Crawley A., Newman A.P., Spencer H., Feldpausch A. (2022). Epidemiologic and Clinical Characteristics of Monkeypox Cases—United States, 17 May–22 July 2022. MMWR Morb. Mortal. Wkly. Rep..

[11] Kava C.M., Rohraff D.M., Wallace B., Mendoza-Alonzo J.L., Currie D.W., Munsey A.E., Roth N.M., Bryant-Genevier J., Kennedy J.L., Weller D.L. (2022). Epidemiologic Features of the Monkeypox Outbreak and the Public Health Response—United States, 17 May–6 October2022. MMWR Morb. Mortal. Wkly. Rep..

[12] Diez Roux A.V. (2002). A glossary for multilevel analysis. J. Epidemiol. Community Health.

[13] Centers for Disease Control and Prevention Mpox Toolkit for People Who Work in Sex Trades or Conduct Outreach to Sex Workers. 23 November 2022. https://archive.cdc.gov/#/details?url=https://www.cdc.gov/poxvirus/mpox/resources/toolkits/sex-workers.html.

[14] Noe S., Zange S., Seilmaier M., Antwerpen M.H., Fenzl T., Schneider J., Spinner C.D., Bugert J.J., Wendtner C.M., Wölfel R. (2023). Clinical and virological features of first human monkeypox cases in Germany. Infection.

[15] Antinori A., Mazzotta V., Vita S., Carletti F., Tacconi D., Lapini L.E., D’Abramo A., Cicalini S., Lapa D., Pittalis S. (2022). Epidemiological, clinical and virological characteristics of four cases of monkeypox support transmission through sexual contact, Italy, May 2022. Euro Surveill..

[16] Safir A., Safir M., Henig O., Nahari M., Halutz O., Levytskyi K., Mizrahi M., Yakubovsky M., Adler A., Ben-Ami R. (2023). Nosocomial transmission of MPOX virus to health care workers -an emerging occupational hazard: A case report and review of the literature. Am. J. Infect. Control.

[17] Choi Y., Jeon E.B., Kim T., Choi S.J., Moon S.M., Song K.H., Kim H.B., Kim E.S. (2023). Case Report and Literature Review of Occupational Transmission of Monkeypox Virus to Healthcare Workers, South Korea. Emerg. Infect. Dis..

[18] Salvato R.S., Ikeda M.L.R., Barcellos R.B., Godinho F.M., Sesterheim P., Bitencourt L.C.B., Gregianini T.S., da Veiga A.B.G., Spilki F.R., Wallau G.L. (2022). Possible Occupational Infection of Healthcare Workers with Monkeypox Virus, Brazil. Emerg. Infect. Dis..

[19] Caldas J.P., Valdoleiros S.R., Rebelo S., Tavares M. (2022). Monkeypox after Occupational Needlestick Injury from Pustule. Emerg. Infect. Dis..

[20] Carvalho L.B., Casadio L.V.B., Polly M., Nastri A.C., Turdo A.C., de Araujo Eliodoro R.H., Sabino E.C., Levin A.S., de Proença A.C.T., Higashino H.R. (2022). Monkeypox Virus Transmission to Healthcare Worker through Needlestick Injury, Brazil. Emerg. Infect. Dis..

[21] Le Pluart D., Ruyer-Thompson M., Ferré V.M., Mailhe M., Descamps D., Bouscarat F., Lescure F.X., Lucet J.C., Yazdanpanah Y., Ghosn J. (2022). A Healthcare-Associated Infection with Monkeypox Virus of a Healthcare Worker During the 2022 Outbreak. Open Forum. Infect. Dis..

[22] Migaud P., Hosmann K., Drauz D., Mueller M., Haumann J., Stocker H. (2023). A case of occupational transmission of mpox. Infection.

[23] Petersen B.W., Kabamba J., McCollum A.M., Lushima R.S., Wemakoy E.O., Tamfum J.J.M., Nguete B., Hughes C.M., Monroe B.P., Reynolds M.G. (2019). Vaccinating against monkeypox in the Democratic Republic of the Congo. Antivir. Res..

[24] Marshall K.E., Barton M., Nichols J., de Perio M.A., Kuhar D.T., Spence-Davizon E., Barnes M., Herlihy R.K., Czaja C.A., Colorado Healthcare Personnel Monitoring Team (2022). Health Care Personnel Exposures to Subsequently Laboratory-Confirmed Monkeypox Patients—Colorado, 2022. MMWR Morb. Mortal. Wkly. Rep..

[25] Shenoy E.S., Wright S.B., Barbeau D.N., Foster L.A., King A.D., Gordon P.S., Mehrotra P., Pepe D.E., Caroff D.A., Kim L.R. (2022). Contact Tracing and Exposure Investigation in Response to the First Case of Monkeypox Virus Infection in the United States During the 2022 Global Monkeypox Outbreak. Ann. Intern. Med..

[26] Szkiela M., Wiszniewska M., Lipińska-Ojrzanowska A. (2023). Monkeypox (Mpox) and Occupational Exposure. Int. J. Environ. Res. Public. Health.

[27] Croft D.R., Sotir M.J., Williams C.J., Kazmierczak J.J., Wegner M.V., Rausch D., Graham M.B., Foldy S.L., Wolters M., Damon I.K. (2007). Occupational risks during a monkeypox outbreak, Wisconsin, 2003. Emerg. Infect. Dis..

[28] Kile J.C., Fleischauer A.T., Beard B., Kuehnert M.J., Kanwal R.S., Pontones P., Messersmith H.J., Teclaw R., Karem K.L., Braden Z.H. (2005). Transmission of monkeypox among persons exposed to infected prairie dogs in Indiana in 2003. Arch Pediatr. Adolesc. Med..

[29] Su C.P., de Perio M.A., Cummings K.J., McCague A.B., Luckhaupt S.E., Sweeney M.H. (2019). Case Investigations of Infectious Diseases Occurring in Workplaces, United States, 2006–2015. Emerg. Infect. Dis..

[30] de Perio M.A., Materna B.L., Sondermeyer Cooksey G.L., Vugia D.J., Su C.P., Luckhaupt S.E., McNary J., Wilken J.A. (2019). Occupational coccidioidomycosis surveillance and recent outbreaks in California. Med. Mycol..

