# Supplementary material for: Analysis of Mpox by Occupation and Industry in Seven U.S. Jurisdictions, May 2022–March 2023
Source: Int J Environ Res Public Health. 2024 Oct 3;21(10):1317. doi: 10.3390/ijerph21101317 (PMC11507617; doi:10.3390/ijerph21101317)
Supplement: Supplementary file 1 [file ijerph-21-01317-s001.zip › ijerph-3186633-supplementary.pdf]

Supplementary Table S1. Occupational data collection details for participating states

State	Range of onset dates for mpox cases included in the analytic sample	Notes on data collection
CA	May 2022 - Oct 2022	All CA reportable disease case report forms include occupation using a combination of dropdown fields and free text fields for specifying occupations not included in the dropdown. Supplemental data on specific occupations and work settings (industry) are collected using a combination of dropdown, checkbox and free text fields. Occupational health data collection for mpox in CA is through the case report form and is encouraged but not required for all local health jurisdictions.
DC	Jun 2022 - Nov 2022	DC Health began collecting case data on 6/3/2022, when the first case was reported. DC Health began collecting occupational data at that time. Occupational data was collected for all interviewed cases as part of the standard interview. Investigators inquired about occupation, employer, and history of onsite work leading up to and during their symptoms, including specific dates worked.
GA	Sep 2022 - Dec 2022	N/A
MA	May 2022 - Nov 2022	Occupation and industry are required data elements for mpox reporting per MA regulation (105 CMR 300) as of 5/27/2022.
MI	Jun 2022 - Nov 2022	For Michigan, the collection of mpox case data, including collecting occupational data from mpox cases, began on 6/8/2022. Occupational data collection is standard for all infectious diseases in Michigan, though it is not required. Training to promote documentation of industry and occupation in the Michigan Disease Surveillance System for infectious disease cases was provided in October 2022.
WA	Jun 2022 - Mar 2023	WA began collecting mpox case data May 25, 2022, based on the CDC required variables, which did not include industry and occupation. Initial electronic collection was done using REDCap and included free-text industry and occupation questions as well as additional employment questions beginning August 12, 2022. Some local health jurisdictions retrospectively entered occupational and employment information. Data collection was moved to Washington Disease Reporting System (Maven) October 20, 2022, to only include industry and occupation free-text (without employer).
WI	Jun 2022 - Oct 2022	Mpox was not a reportable condition in Wisconsin before the outbreak, however “unusual diseases” are always reportable so WI received reports under that mechanism. The first suspect cases were reported into the WI electronic disease system on June 6, 2022. Industry and occupation are routinely collected in WI's reportable disease surveillance system and were included in WI's original mpox CRF.

Supplementary Table S2. Breakdown of state-specific contributions of mpox cases to the analytic sample

STATE	Total mpox cases reported to CDC as of 23 Mar 2023	Cases Reported to NIOSH as of 23 Mar 2023[1]	Percent of all Cases Reported to NIOSH	Reported cases with valid occupation or industry code assigned	Percent of all cases with valid occupation or industry code assigned	Percent of reported cases with valid occupation or industry code assigned	Proportion of total analytic sample	Reported Cases with Valid Occupation Code	Percent of All Cases with Valid Occupation Code	Percent of Reported Cases with Valid Occupation Code	Reported Cases with Valid Industry Code	Percent of All Cases with Valid Industry Code	Percent of Reported Cases with Valid Industry Code
CA	5746	5135	89.37%	1547	26.92%	30.13%	60.71%	1266	22.03%	24.65%	1334	23.22%	25.98%
DC	527	524	99.43%	242	45.92%	46.18%	9.50%	225	42.69%	42.94%	229	43.45%	43.70%
GA	1993	37	1.86%	30	1.51%	81.08%	1.18%	29	1.46%	78.38%	29	1.46%	78.38%
MA	460	456	99.13%	284	61.74%	62.28%	11.15%	243	52.83%	53.29%	257	55.87%	56.36%
MI	399	381	95.49%	147	36.84%	38.58%	5.77%	110	27.57%	28.87%	141	35.34%	37.01%
WA	687	266	38.72%	251	36.54%	94.36%	9.85%	212	30.86%	79.70%	233	33.92%	87.59%
WI	88	86	97.73%	47	53.41%	54.65%	1.84%	42	47.73%	48.84%	46	52.27%	53.49%
Total	9900	6885	69.55%	2548	25.74%	37.01%	100.00%	2127	21.48%	30.89%	2269	22.92%	32.96%

[1] Number of cases from which occupational data could potentially have been collected.

Supplementary Table S3. Proportionate morbidity ratios (PMRs) and 99% confidence intervals (CIs) by occupation group and gender in a sample of employed mpox cases from seven U.S. jurisdictions, May 2022 - March 2023

	Overall					Men					Women				
				99% CI					99% CI					99% CI	
**Occupational group**	**Observed Cases**	**Expected Cases**	**PMR**	**LCL**	**UCL**	**Observed Cases**	**Expected Cases**	**PMR**	**LCL**	**UCL**	**Observed Cases**	**Expected Cases**	**PMR**	**LCL**	**UCL**
MANAGEMENT	197	283.23	*0.70*	*0.57*	*0.83*	186	288.97	*0.64*	*0.53*	*0.77*	8	7.38	1.08	0.32	2.30
BUSINESS & FINANCIAL OPERATIONS	102	132.22	*0.77*	*0.59*	*0.98*	100	109.36	0.91	0.69	1.17	0	4.34	--	--	--
COMPUTER & MATHEMATICAL	79	110.73	*0.71*	*0.52*	*0.94*	78	144.09	*0.54*	*0.39*	*0.71*	1	1.85	0.54	0.04	2.84
ARCHITECTURE & ENGINEERING	42	54.29	0.77	0.50	1.11	42	77.67	*0.54*	*0.35*	*0.78*	0	0.67	--	--	--
LIFE, PHYSICAL, & SOCIAL SCIENCE	48	43.25	1.11	0.74	1.56	48	40.35	1.19	0.79	1.67	0	1.26	--	--	--
COMMUNITY & SOCIAL SERVICES	33	41.71	0.79	0.48	1.19	32	25.35	1.26	0.75	1.90	0	1.64	--	--	--
LEGAL	23	36.66	0.63	0.34	1.01	22	32.64	0.67	0.35	1.09	0	1.20	--	--	--
EDUCATION, TRAINING, & LIBRARY	94	114.43	0.82	0.62	1.05	89	58.45	**1.52**	**1.14**	**1.97**	5	4.77	1.05	0.19	2.60
ARTS, DESIGN, ENTERTAINMENT, SPORTS, & MEDIA	81	67.73	1.20	0.88	1.56	81	68.31	1.19	0.87	1.55	0	1.79	--	--	--
HEALTHCARE PRACTITIONERS & TECHNICAL	135	111.78	1.21	0.95	1.49	126	55.23	**2.28**	**1.79**	**2.83**	9	4.74	1.90	0.62	3.88
HEALTHCARE SUPPORT	58	71.01	0.82	0.56	1.12	54	24.84	**2.17**	**1.48**	**3.00**	4	3.41	1.17	0.15	3.17
PROTECTIVE SERVICE	27	39.03	0.69	0.39	1.08	27	52.48	*0.51*	*0.29*	*0.80*	0	0.59	--	--	--
FOOD PREPARATION & SERVING RELATED	194	111.53	**1.74**	**1.43**	**2.08**	191	98.98	**1.93**	**1.59**	**2.31**	3	3.42	0.88	0.06	2.67
BUILDING & GROUNDS CLEANING & MAINTENANCE	90	72.92	1.23	0.92	1.59	89	77.39	1.15	0.86	1.49	1	1.83	0.55	0.05	2.87
PERSONAL CARE & SERVICE	113	53.20	**2.12**	**1.64**	**2.67**	106	23.88	**4.44**	**3.40**	**5.62**	7	2.39	2.93	0.77	6.47
SALES & RELATED	212	167.78	**1.26**	**1.05**	**1.50**	209	145.62	**1.44**	**1.19**	**1.70**	3	5.27	0.57	0.04	1.73
OFFICE & ADMINISTRATIVE SUPPORT	206	185.14	1.11	0.92	1.32	195	109.54	**1.78**	**1.47**	**2.12**	10	7.53	1.33	0.47	2.63
FARMING, FISHING, & FORESTRY	3	21.70	*0.14*	*0.01*	*0.42*	3	26.81	*0.11*	*0.01*	*0.34*	0	0.41	--	--	--
CONSTRUCTION & EXTRACTION	72	109.13	*0.66*	*0.47*	*0.88*	72	186.11	*0.39*	*0.28*	*0.51*	0	0.30	--	--	--
INSTALLATION, MAINTENANCE, & REPAIR	22	48.66	*0.45*	*0.24*	*0.73*	22	82.77	*0.27*	*0.14*	*0.43*	0	0.15	--	--	--
PRODUCTION	61	89.21	*0.68*	*0.48*	*0.93*	57	105.85	*0.54*	*0.37*	*0.74*	4	1.86	2.15	0.27	5.82
TRANSPORTATION & MATERIAL MOVING	214	140.74	**1.52**	**1.26**	**1.80**	211	194.32	1.09	0.90	1.29	3	1.98	1.51	0.10	4.60
ARMED FORCES	21	20.93	1.00	0.52	1.65	20	30.98	0.65	0.33	1.07	1	0.24	4.18	0.35	21.90
Total	2127	2127.00	1.00			2060	2060.00	1.00			59	59.00	1.00		

Values in *italics* indicate PMRs that are significantly (p<0.01) lower than expected.
Values in **bold** indicate PMRs that are significantly (p<0.01) higher than expected.

Supplementary Table S4. Proportionate morbidity ratios (PMRs) and 99% confidence intervals (CIs) for by industry group and gender in a sample of employed mpox cases from seven U.S. jurisdictions, May 2022 - March 2023

	Overall					Men					Women				
	Observed	Expected		99% CI		Observed	Expected		99% CI		Observed	Expected		99% CI	
Industry sector	Cases	Cases	PMR	LCL	UCL	Cases	Cases	PMR	LCL	UCL	Cases	Cases	PMR	LCL	UCL
AGRICULTURAL, FORESTRY, FISHING & HUNTING	5	34.07	*0.15*	*0.03*	*0.36*	5	43.63	*0.11*	*0.02*	*0.28*	0	0.62	--	--	--
MINING	0	2.26	0.00	*0.74*	*0.74*	0	3.36	0.00	*0.49*	*0.49*	0	0.02	--	--	--
CONSTRUCTION	84	162.11	*0.52*	*0.38*	*0.67*	84	257.77	*0.33*	*0.24*	*0.42*	0	1.08	--	--	--
DURABLE GOODS MANUFACTURING	76	125.80	*0.60*	*0.44*	*0.80*	74	164.09	*0.45*	*0.33*	*0.60*	2	2.16	0.93	0.01	3.38
NONDURABLE GOODS MANUFACTURING	67	58.94	1.14	0.81	1.52	65	64.12	1.01	0.72	1.36	2	1.46	1.37	0.01	4.99
WHOLESALE TRADE	18	36.84	*0.49*	*0.24*	*0.83*	18	45.27	*0.40*	*0.19*	*0.68*	0	0.72	--	--	--
RETAIL TRADE	228	208.66	1.09	0.91	1.29	220	188.77	1.17	0.97	1.38	8	6.45	1.24	0.37	2.63
TRANSPORTATION & WAREHOUSING	149	113.45	**1.31**	**1.05**	**1.61**	147	149.13	0.99	0.79	1.21	1	1.88	0.53	0.04	2.78
UTILITIES	6	17.91	*0.34*	*0.08*	*0.78*	6	24.61	*0.24*	*0.05*	*0.57*	0	0.26	--	--	--
INFORMATION	36	62.03	*0.58*	*0.36*	*0.86*	34	71.13	*0.48*	*0.29*	*0.71*	2	1.40	1.43	0.01	5.22
FINANCE & INSURANCE	52	83.05	*0.63*	*0.42*	*0.87*	51	75.14	*0.68*	*0.46*	*0.95*	1	2.56	0.39	0.03	2.05
REAL ESTATE & RENTAL & LEASING	52	45.82	1.13	0.77	1.58	51	44.32	1.15	0.77	1.60	0	1.33	--	--	--
PROFESSIONAL & TECHNICAL SERVICES	230	263.99	0.87	0.73	1.03	225	274.35	*0.82*	*0.69*	*0.97*	3	6.82	0.44	0.03	1.34
MANAGEMENT, ADMINISTRATIVE & WASTE MANAGEMENT SERVICES	93	98.58	0.94	0.71	1.21	93	105.46	0.88	0.66	1.13	0	2.54	--	--	--
EDUCATIONAL SERVICES	124	188.88	*0.66*	*0.51*	*0.82*	121	107.60	1.12	0.88	1.40	3	7.90	0.38	0.02	1.15
HEALTH CARE & SOCIAL ASSISTANCE	407	279.83	**1.45**	**1.27**	**1.65**	383	122.14	**3.14**	**2.74**	**3.56**	23	12.97	1.77	0.95	2.85
ARTS, ENTERAINMENT & RECREATION	101	61.11	**1.65**	**1.26**	**2.10**	98	54.56	**1.80**	**1.36**	**2.29**	3	1.92	1.56	0.10	4.75
ACCOMMODATION & FOOD SERVICES	278	155.64	**1.79**	**1.52**	**2.07**	271	144.06	**1.88**	**1.60**	**2.19**	7	4.71	1.49	0.39	3.29
PRIVATE HOUSEHOLDS	9	12.77	0.70	0.23	1.44	8	2.50	3.20	0.95	6.78	1	0.69	1.45	0.12	7.62
OTHER SERVICES, EXCEPT PRIVATE HOUSEHOLDS	165	105.28	**1.57**	**1.27**	**1.90**	155	98.99	**1.57**	**1.26**	**1.91**	6	3.18	1.89	0.42	4.39
PUBLIC ADMINISTRATION	68	130.16	*0.52*	*0.37*	*0.70*	66	121.76	*0.54*	*0.38*	*0.73*	2	4.06	0.49	0.00	1.80
ARMED FORCES	21	21.81	0.96	0.50	1.58	20	32.24	0.62	0.31	1.03	1	0.27	3.70	0.31	19.36
Total	2269	2269.00	1.00			2195	2195.00	1.00			65	65.00	1.00		

Values in *italics* indicate PMRs that are significantly (p<0.01) lower than expected.

Values in **bold** indicate PMRs that are significantly (p<0.01) higher than expected.

Supplementary Table S5. Most frequently reported detailed occupations within identified high-risk occupation groups in a sample of employed mpox cases among men from seven U.S. jurisdictions, May 2022 - March 2023

Detailed occupation	No.	%*
**Personal care and service occupations (n=106)**		
Hairdressers, Hairstylists, and Cosmetologists	33	31.13
Personal Care Aides	17	16.04
Recreation and Fitness Workers	14	13.21
Personal Care and Service Workers, All Other	11	10.38
Nonfarm Animal Caretakers	9	8.49
**Healthcare practitioners and technical occupations (n=126)**		
Registered Nurses	45	35.71
Physicians and Surgeons	17	13.49
Health Practitioner Support Technologists and Technicians	12	9.52
Clinical Laboratory Technologists and Technicians	10	7.94
Dentists	6	4.76
**Healthcare support occupations (n=54)**		
Nursing, Psychiatric, and Home Health Aides	20	37.04
Massage Therapists	14	25.93
Medical Assistants	8	14.81
Nursing, Psychiatric, and Home Health Aides	5	9.26
Dental Assistants	2	3.70
**Food preparation and serving related occupations (n=191)**		
Waiters and Waitresses	47	24.61
Cooks	41	21.47
Bartenders	31	16.23
First-Line Supervisors of Food Preparation and Serving Workers	20	10.47
Food Preparation Workers	19	9.95
**Office and administrative support occupations (n=195)**		
Secretaries and Administrative Assistants	49	25.13
Office Clerks, General	44	22.56
Receptionists and Information Clerks	16	8.21
Office and Administrative Support Workers, All Other	11	5.64
Stock Clerks and Order Fillers	11	5.64
**Education, training, and library occupations (n=89)**		
Elementary and Middle School Teachers	30	33.71
Other Teachers and Instructors	17	19.10
Postsecondary Teachers	12	13.48
Other Education, Training, and Library Workers	10	11.24
Preschool and Kindergarten Teachers	7	7.87
**Sales and related occupations (n=209)**		
Retail Salespersons	98	46.89
Sales and Related Workers, All Other	28	13.40
Cashiers	21	10.05
First-Line Supervisors of Retail Sales Workers	21	10.05
Real Estate Brokers and Sales Agents	18	8.61

*Percent of occupation group, not overall sample

Supplementary Table S6. Most frequently reported detailed occupations within identified high-risk industry sectors in a sample of employed mpox cases among men from seven U.S. jurisdictions, May 2022 - March 2023

Detailed occupation	No.	%*
**Health care and social assistance industry (n=230)**		
Registered Nurses	40	17.39
Nursing, Psychiatric, and Home Health Aides	21	9.13
Physicians and Surgeons	15	6.52
Personal Care Aides	11	4.78
Secretaries and Administrative Assistants	8	3.48
**Accommodation and food services industry (n=242)**		
Waiters and Waitresses	46	19.01
Cooks	38	15.70
Bartenders	31	12.81
First-Line Supervisors of Food Preparation and Serving Workers	19	7.85
Food Preparation Workers	18	7.44
**Arts, entertainment, and recreation industry (n=58)**		
Recreation and Fitness Workers	9	15.52
Musicians, Singers, and Related Workers	7	12.07
Writers and Authors	5	8.62
Dancers and Choreographers	4	6.90
Managers, All Other	4	6.90
**Other services, except private households (n=134)**		
Hairdressers, Hairstylists, and Cosmetologists	33	24.63
Massage Therapists	13	9.70
Personal Care and Service Workers, All Other	10	7.46
Barbers	8	5.97
Nonfarm Animal Caretakers	8	5.97

*Percent of industry sector (excluding cases with missing occupation data), not overall sample

Supplementary Table S7. Most frequently reported detailed industries in identified high-risk industry sectors in a sample of employed mpox cases among men from seven U.S. jurisdictions, May 2022 - March 2023

	No.	%*
**Health care and social assistance industry (n=383)**		
Outpatient Care Centers	162	42.30
Hospitals	104	27.15
Nursing Care Facilities (Skilled Nursing Facilities)	31	8.09
Individual and Family Services	18	4.70
Offices of Physicians	12	3.13
**Accommodation and food services industry (n=271)**		
Restaurants and Other Food Services	208	76.75
Traveler Accommodation	33	12.18
Drinking Places, Alcoholic Beverages	29	10.70
Recreational Vehicle Parks and Camps, and Rooming and Boarding Houses	1	0.37
**Arts, entertainment, and recreation industry (n=98)**		
Performing Arts, Spectator Sports, and Related Industries	65	66.33
Other Amusement, Gambling, and Recreation Industries	28	28.57
Museums, Art Galleries, Historical Sites, and Similar Institutions	5	5.10
**Other services, except private households (n=155)**		
Beauty Salons	37	23.87
Other Personal Services	35	22.58
Nail Salons and Other Personal Care Services	27	17.42
Civic, Social, Advocacy Organizations, and Grantmaking and Giving Services	23	14.84
Barber Shops	7	4.52

*Percent of industry sector, not overall sample
